# Lethal Effects of High Temperatures on Brown Marmorated Stink Bug Adults before and after Overwintering

**DOI:** 10.3390/insects10100355

**Published:** 2019-10-18

**Authors:** Davide Scaccini, Carlo Duso, Alberto Pozzebon

**Affiliations:** Department of Agronomy Food Natural Resources Animals and Environment, University of Padova, 35020 Legnaro, Padova, Italy; davide.scaccini@phd.unipd.it (D.S.); carlo.duso@unipd.it (C.D.)

**Keywords:** rapid heating, temperature tolerance, *Halyomorpha halys*, quarantine, nutrient index, heat treatment, diapause

## Abstract

The invasive brown marmorated stink bug, *Halyomorpha halys*, is causing economic and ecological damage in invaded areas. Its overwintering behavior warrants mitigation practices in warehouses and shipping operations. The aim of this study was to characterize the mortality response curves of *H. halys* adults to short high-temperature exposure. Here we compared field-collected individuals entering (ENA) and exiting diapause (EXA). EXA adults displayed increased susceptibility to high temperatures compared to ENA individuals. Complete mortality of all tested individuals was obtained after 10 min exposure at 50.0 °C, and after 15 (EXA) or 20 min (ENA) at 47.5 °C. The nutritional status of these insects had no effect on high-temperature tolerance. The mortality curves obtained here may be used for the definition of cost-effective heat treatments aimed at the *H. halys* control.

## 1. Introduction

Invasive insect species cause significant economic and ecological damage in newly-invaded areas [1,2], resulting in high economic costs [3,4]. In the recent past, the brown marmorated stink bug, *Halyomorpha halys* (Stål) (Hemiptera: Pentatomidae) started to spread worldwide from its native range in East Asia. It is an invasive phytophagous pest, already recorded to feed on more than 170 plant species worldwide [5]. In Europe, the first *H. halys* was recorded outside its native range in the 2000s in Switzerland [6], and it is now widespread throughout many European countries [5,7,8]. In Italy, *H. halys* rapidly became an agricultural pest, resulting in damage to crops and causing nuisance problems for the human population [9,10,11].

In autumn, adult *H. halys* field populations invade buildings searching for shelters in which to overwinter [12,13]. As a result, adults can be found in warehouses and in food industries that are present in the area of invasion, with potential risks of the contamination of goods that could be distributed worldwide [14,15]. This aspect plays a key role in the invasion process of this pest. Overwintering aggregations are frequently intercepted in freightliners transporting cargo, and this is likely to represent an important invasion pathway for *H. halys* worldwide [7,16,17].

In disinfestation programs, insect control in food industries can be achieved by modifying the environmental temperature below or above insects’ critical temperature levels. The use of extreme temperatures can be a viable method in quarantine security in order to avoid the introduction of exotic pests into other countries. Treatments using suboptimal low temperatures (i.e., freezing) are less commonly used due to the high energetic costs, while high temperatures have been extensively used since the last century [18,19,20,21] to kill insect pests of stored products [22,23]. Furthermore, heat treatments have been proposed for the control of other insects infesting commodities and against structural pests, where killing temperatures can be applied in multiple forms, such as hot water, vapor heat, forced hot air, dry heat, or other methodologies [24,25,26].

Acute changes in temperature result in significant metabolic changes in insects. These changes affect many physiological processes, including anaerobiotic metabolism, enzyme activity, altered cellular membranes, behavior, and nervous and endocrine systems. External structures such as the cuticular wax of the insect can also be modified [27]. Above 40.0 °C these effects become more critical [27]. Generally, short exposures up to temperatures of 50.0 °C are lethal for almost all tested insects [28]. Similar high-temperature treatments are used for quarantine treatments of goods [29], where exposures of 50–60 °C for 24 h are required in disinfestation programs [23].

For *H. halys*, it is known that consistently low temperature is required to kill adults [30,31,32], making cold treatments a less suitable practice in disinfestation programs. Conversely, heat treatments have been proposed to manage *H. halys* in closed environments by exposing goods possibly containing insects to 50.0 °C for 15 min or more [33]. However, more detailed information is necessary to optimize heat treatments, in order to reduce energetic costs and increase efficacy in *H. halys* control.

Additionally, treatments aimed at managing “hitchhiking” pest populations within shipped goods may involve *H. halys* in different physiological states. *Halyomorpha halys* can overwinter as aggregations of adults in non-feeding and non-reproductive state within hidden and protected sites [34,35]. Such insects typically display gradual springtime emergence and field dispersion patterns. This transition from overwintering to the overwintered state is associated with non-reproductive to reproductive physiological changes [36,37]. When overwintering, the nutritional levels of *H. halys* populations decline [38] but then display a gradual increase as the winter progresses through the summer [39]. Different physiological and nutritional statuses can have a potential effect on resistance to high temperatures, however this information is not available for *H. halys* since previous research was performed using laboratory-maintained colonies [33].

The aim of this study was to characterize short-term (2.5 min to 1 h) high-temperature mortality response curves of *H. halys* adults and to test if the effect of heat exposure changes between adults entering and exiting diapause. Lastly, we tested whether the nutritional status of *H. halys* influenced their mortality induced by heat exposure.

## 2. Materials and Methods

### 2.1. Insects

Both entering diapause (September to October; hereafter ENA) and exiting diapause adults (April to May; hereafter EXA) were hand-collected in Legnaro, Italy (45.344872 N, 11.956208 E) in 2017. ENA adults were insects that developed in the current year (two generations per year are recorded in northern Italy [40]). EXA adults were collected from artificial overwintering units placed in outdoor conditions under shade and covered to be protected from weather events such as rainfall. Artificial overwintering units consisted of five plastic boxes (50 × 35 × 15 cm^3^; IKEA^®^, Delft, The Netherlands) containing wooden cages (34 × 19 × 10 cm^3^) with a 34 × 1 cm^2^ slit along one side. Cardboard and folded paper (abt. 20 × 20 cm^2^) were placed inside wooden cages to provide shelter for insects. In fall 2016, 250 *H. halys* adults were placed in each box. These units were monitored three times per week from November 2016 to May 2017. Adults were considered to be exiting diapause when they were found outside the wooden cages in the plastic boxes. Diapause was confirmed by insect dissection, where no eggs were recorded in ENA or EXA females which were in the “one immature oocyte per ovariole” rank as described by Nielsen et al. [37].

### 2.2. Lethal High Temperatures with Short Exposure Times

Laboratory experiments were performed using a thermocryostat (LAUDA Alpha, RA 12^®^, LAUDA DR. R. WOBSER GMBH & CO. KG, Lauda-Königshofen, Germany) for heat treatment. *Halyomorpha halys* adults were placed singly in glass vials (7-mL volume) and were sealed by a cotton swab to allow gas exchange. The available air volume of vials for each adult was about 5.5 mL. The starting relative humidity was 50(± 2)%, and fluctuated to 56(± 4)% during the experiment. At least 30 adults per temperature-time combination were tested, in replicates of 10 vials. Before treatment, adults were collected from buildings (ENA) or from overwintering units (EXA) and kept for two hours in boxes at room temperature (23 °C). Vials were placed in the thermocryostat and exposed to temperatures ranging from 32.5 to 60.0 °C, with a 2.5 °C step (i.e., 12 temperature levels: 32.5, 35.0, 37.5, 40.0, 42.5, 45.0, 47.5, 50.0, 52.5, 55.0, 57.5, or 60.0 °C, herein referred to as set temperatures), for six different time periods (i.e., 2.5, 5, 10, 15, 30, or 60 min) for both ENA and EXA adults. The following six temperatures were also performed only for the ENA adults: 41.5, 45.5, 46.0, 46.5, 47.0, and 56.5 °C. The temperature within the vials was checked using a thermocouple (RhOS, 4-Channel Digital Thermometer Thermocouple Sensor). Heating rate was 15.0 °C min^−1^, with heating times ranging from 0.6 to 2.5 min depending on the set temperature. When the set temperature was reached, insects were transferred into vials and then placed in the thermocryostat. Vials were kept in the thermocryostat for the time required (i.e., 2.5, 5, 10, 15, 30 or 60 min) according to the experimental design. Then, the insects were removed from vials, kept in rearing cages (30 × 30 × 30 cm^3^; BugDorm-1, MegaView Science Co., Ltd., Taiwan) at room temperature and followed for 24 h to assess the mortality. Control ENA and EXA insects were subjected to room temperature (maintained at 23 °C during all the procedures).

### 2.3. Nutrient Index and *H. halys* Mortality after High-Temperature Exposures

Prior to heat exposure, we evaluated the weight and nutritional level of each individual calculating the nutrient index (NI) as described by Funayama [41] and Skillman et al. (i.e., weight of insect (mg)/prothorax width (mm)^3^ [38,39]). The nutrient index is a non-destructive and easy-to-use method that was used as an indicator of the nutrient status of *H. halys* [41]. We tested if the weight or level of the nutrient index had an effect on adult mortality after heat exposure. Another set of 240 ENA and EXA females and males were exposed to two of the temperatures reported before for 15, 30, or 60 min using the procedures described above. After heat treatment, insects were processed following the same procedure described above.

### 2.4. Data Analysis

Data on the effect of lethal high temperatures were analyzed with a probit regression using the PROBIT procedure of SAS^®^ (ver. 9.4) [42] and interpolating the observed data to mortality curves. Lethal temperatures for 50% (LT50) and 99% (LT99) mortality of adults, for any exposure time, were determined. We ran probit regression on data obtained with 2.5, 15, 30, and 60 min exposure times. Comparisons between the two groups of adults (i.e., EXA and ENA) for the lethal temperature levels (i.e., LT50 and LT99) were done by using the lethal dose ratios method (α = 0.05), which is based on the 95% confidence limits of lethal temperature levels, depending on the intercepts and the slopes of the probit lines and considering the variance-covariance matrices as described by Robertson et al. [43]. The method is used for comparisons of point estimates in different insect mortality curves obtained from treatment exposures [43].

The time causing mortality (MT) was studied through the probit regression analysis for 47.5 and 50.0 °C using all exposure times. The probit analysis was conducted as stated before. In this case, the exposure time was considered as the independent variable and the mortality as the dependent one. Comparisons between the two groups of adults (i.e., EXA and ENA) for the lethal time levels MT50 and MT99 were done using the lethal dose ratios method (α = 0.05) [43].

Finally, data on nutrient index and insect weight were analyzed separately using a general linear mixed model, with the MIXED procedure of SAS^®^ (ver. 9.4) [42] with an F-test (α = 0.05) followed by a Tukey–Kramer test (α = 0.05) to determine if there were differences in nutrient index or weight in dead and alive insects after exposure to 42.5 or 45.0 °C for 15, 30, or 60 min. In this analysis, we considered the status of the insect (i.e., dead or alive), sex, state of diapause (i.e., EXA or ENA), temperature (i.e., 42.5 or 45.0 °C), and their interactions as sources of variation. Data on mortality after 15, 30, or 60 min were analyzed separately. Data were checked for model assumptions prior to the analysis, and untransformed data were used.

## 3. Results

### 3.1. Lethal High Temperatures

**Figure 1:**
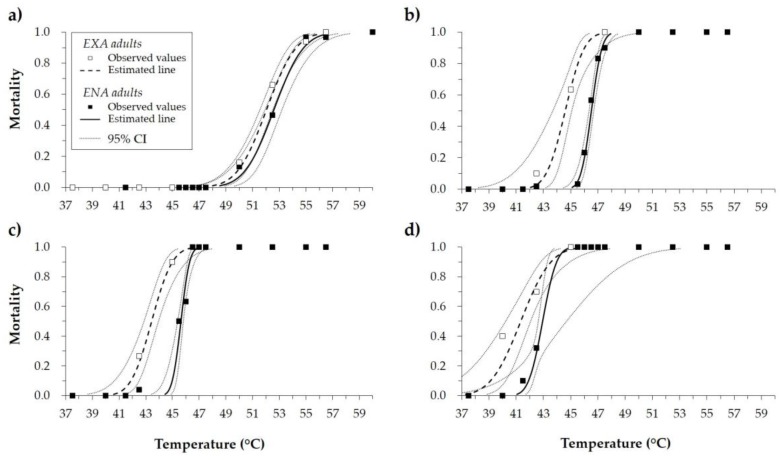
Mortality rate (observed values, estimates of the curves, and 95% confidence interval (CI)) of *Halyomorpha halys* adults (exiting diapause (EXA) and entering diapause (ENA)) after exposure to high temperatures for (**a**) 2.5, (**b**) 15, (**c**) 30, and (**d**) 60 min.

An increase in insect mortality was directly correlated with an increase in temperature. Shorter exposure times required higher temperatures to result in comparable *H. halys* mortality levels. With 2.5 min exposure, the minimum temperature to induce *H. halys* mortality was 50.0 °C for both EXA and ENA adults (Figure 1a). With exposure times of 15 and 30 min, the minimum temperature inducing mortality was 42.5 °C (Figure 1b,c), while after 60 min of exposure the minimum temperature to cause mortality was 40.0 °C or 41.5 °C for EXA and ENA adults, respectively (Figure 1d).

Considering all the exposure times tested, LT50 ranged from 41.3 to 52.6 °C and LT99 from 44.8 to 56.7 °C. Mortality curves were different between EXA and ENA adults. At the same exposure time, LTs were lower for EXA adults, which were more susceptible to high temperatures than ENA adults (Table 1).

### 3.2. Exposure Times at High Temperatures

**Figure 2:**
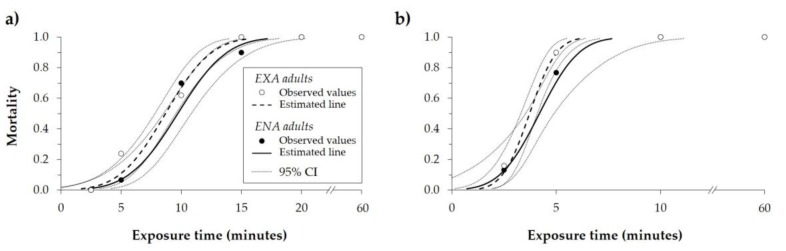
Mortality rate (observed values, estimates of the curves, and 95% confidence interval (CI)) of *Halyomorpha halys* adults (EXA and ENA) calculated after different exposure times at the constant temperature of (**a**) 47.5 °C and (**b**) 50.0 °C.

The lowest time inducing *H. halys* mortality was 2.5 min when adults were exposed to 50.0 °C, and 5 min when exposed to 47.5 °C. The mortality rate was 100% after 10 min of exposure at 50.0 °C, and after 15 (EXA) or 20 min (ENA) at 47.5 °C. No survival was recorded for adults exposed to these two temperatures for 20 min or more (Figure 2). At 47.5 °C, the time required to kill 50% of the adults (MT50) was 8.67 and 9.69 min, while the MT99 was 15.65 and 17.14 min for EXA and ENA, respectively (Table 2). At 50 °C, MT50 was 3.72 and 4.19 min, while the MT99 was 6.10 and 7.67 min for EXA and ENA, respectively (Table 2). For the exposure to the same temperature, the lethal dose ratios method did not show differences between the EXA and ENA curves for the MT50, while MT99 was higher for ENA than EXA at both tested temperatures (Table 2).

### 3.3. Weight and Nutrient Index

The mortality rate of *H. halys* after exposure to 42.5 and 45.0 °C for 15, 30, and 60 min was not different considering insect weight or nutrient index (Table 3 and Table 4). Weight (Table 3) and nutrient index values (Table 4) were higher for ENA (weight: 137.36 ± 36.99 mg; NI: 0.24 ± 0.03) than for EXA (weight: 115.27 ± 26.76 mg; NI: 0.20 ± 0.03) adults, and the weight was higher for females (147.83 ± 32.35 mg) than for males (105.91 ± 20.38 mg; Table 3 and Table 4).

## 4. Discussion

The mortality parameters from different time and temperature combinations provide significant new insights into effective heat treatments aimed at *H. halys* control. These data deliver scenarios where high levels of *H. halys* mortality were obtained using relatively limited energetic costs. Such heat treatments can be effectively used as part of quarantine procedures for *H. halys* contamination. Both ENA and EXA adults are likely *H. halys* life stages that could be effectively targeted by such heat treatments. These insects are in a non-reproductive state [36,37], as confirmed by the absence of eggs in ENA and EXA females in our study. ENA adults enter into overwintering sites in autumn, while EXA adults emerge from these sites in spring [40]. In general, these *H. halys* adults do not have to cope with high-temperature stress because such high temperatures do not normally occur in autumn or spring. On the other hand, adults developed during the warmer period of the year (which move to overwintering sites at the end of the summer [12,13]) may have some forms of tolerance to high temperatures. This topic fell outside the scope of this study and should be investigated in future.

*Halyomorpha halys* adults start to emerge from overwintering sites when temperatures exceed 10.0 °C, and flight activity strongly increases at temperatures above 15.0 °C [44,45,46]. In shipping cargo, temperature up to 30 °C could stimulate the activity of overwintering *H. halys*, but these insects will die due to the lack of available food [17]. Aigner and Kuhar [33] found that a minimum temperature of 45.0 °C for 15 min or 35.0 °C for 4 h were required to kill *H. halys* adults in the laboratory, but a precise minimum threshold could not be derived from their data. Additionally, they used *H. halys* from an artificial mass rearing colony and did not assess potential effects of adult diapause state. In the present manuscript we observed that mortality of *H. halys* started at 40.0 °C and 41.5 °C for 60 min for EXA and ENA adults, respectively. Using data from probit regression we determined a minimum threshold for *H. halys* mortality that was 41.1 °C for ENA adults and 37.3 °C for EXA adults considering a 60 min exposure, while higher thresholds were obtained with 2.5 min of exposure (i.e., 48.6 °C and 48.0 °C for ENA and EXA adults, respectively).

Notably, our results showed that EXA adults were more sensitive to high temperatures than ENA ones, possibly due to the physiological status of the insect, as adults entering diapause have a reduction of nutritional levels and a different energetic fitness than adults exiting diapause [38,39]. EXA adults had a lower weight and nutrient index than ENA adults. However, the lipid, glycogen, and sugar content do not seem to be related to the nutrient index of *H. halys* adults [38]. In our study, the state of diapause well explained the mortality levels showed by *H. halys* adults, while the nutrient index or the weight did not, confirming that the adult’s state of diapause more than their nutrient index should be considered during the optimization of heat treatment against *H. halys*.

Furthermore, all the tested *H. halys* adults died with exposures to at least 48.0 °C for 15 min, or 45.5 °C for 1 h. In the USA, *H. halys* high-temperature mortality showed no adult survival with the exposure to 50.0 °C for at least 15 min, or to 45.0 °C for 1 h or more [33]. Here we found that the time required to kill 99% of the adults was ~17 or ~7.5 min for 47.5 or 50 °C, respectively (considering ENA adults as the most conservative case), highlighting the temperature–time combinations that may be considered for practical uses for heat treatment at quarantine facilities. Differences on mortality detected between studies may be related to the use of laboratory-reared insects (as in Aigner and Kuhar [33]) versus field-collected ones (as in this study), but also by genetic features of the tested insects [47] or the methodology performed [48,49,50].

The results of the present study, and in particular data on minimum thresholds for *H. halys* mortality obtained with 1-h exposure, can also be of importance in forecasting the geographical distribution of this invasive pest. Previous published data on *H. halys* development also showed that European populations failed to develop under controlled conditions at 35.0 °C or more [51], implying possible restrictions in the distribution area of the species and influencing its phenology in areas where unsuitable climate conditions and heatwaves occur [52]. Heatwaves, which are extreme short-term climatic events defined as prolonged periods of excessive heat, are globally increasing in frequency [53,54]. Typically, during heatwaves in several parts of the word, maximum daily temperatures can exceed 35–40.0 °C for several days [55,56,57]. This temperature range poses risks for the survival of *H. halys* in some of these areas (e.g., southern Europe and Australia) where heatwaves can be associated with temperatures above 35.0 °C for hours. Here we found that temperatures higher than 37.3 °C for 1 h could reduce the survival of *H. halys* adults. This type of information can be used to update current models on the dynamics and geographic distribution of this pest [33,58,59,60] accounting for the effect of high environmental temperatures on mortality. Data provided here are not complete for understanding the impact of high environmental temperature on *H. halys*, but may represent a starting point for future research that should investigate the effect of high temperatures on young stages and simulating longer exposure time.

## 5. Conclusions

The results obtained here underline the ability of *H. halys* to tolerate high temperatures. These data provide important parameters that can be used as heat shock treatments for *H. halys* control in quarantine methods for the disinfestation of goods and in fresh food export industries. Heat treatments with short-time exposure should be targeted on the *H. halys* physiological status, which seems to be related to the overwintering state. The definition of cost-effective heat treatments aimed at the *H. halys* control may also be performed following the mortality curves reported here.

## Figures and Tables

**Table 1:** Lethal high temperatures (LT) 50 and 99 with 95% confidence interval (CI) for *Halyomorpha halys* adults of the two investigated states (i.e., ENA and EXA), with probit regression parameters.

Adults’ State	Exposure Time (Minutes)	n	LT50 (°C) *		95% CI_LT50_ (°C)		LT99 (°C) *		95% CI_LT99_ (°C)		Intercept	SE_Intercept_	Slope	SE_Slope_	Χ^2^ **	df
					Lower	Upper			Lower	Upper						
EXA	2.5	310	52.04	b	51.54	52.50	56.07	b	55.22	57.39	−30.01	3.70	0.58	0.07	32.94	29
ENA	2.5	400	52.61	a	51.94	53.20	56.66	a	55.68	58.30	−73.15	4.51	1.55	0.09	26.16	35
EXA	15.0	310	44.60	b	43.69	45.09	47.17	b	46.33	49.81	−40.47	11.49	0.91	0.25	8.77	29
ENA	15.0	400	46.53	a	46.36	46.69	47.95	a	47.62	48.51	−76.34	10.92	1.64	0.23	24.66	35
EXA	30.0	310	43.44	b	42.86	43.96	46.29	b	45.44	48.01	−35.44	6.81	0.82	0.16	10.97	29
ENA	30.0	400	45.64	a	45.38	45.81	46.83	a	46.52	47.53	−89.54	19.04	1.96	0.41	14.17	35
EXA	60.0	310	41.34	b	40.23	42.05	45.39	a	44.26	47.94	−23.74	5.31	0.57	0.13	11.01	29
ENA	60.0	400	42.95	a	42.62	44.70	44.83	b	43.78	53.05	−53.09	20.83	1.24	0.49	4.61	35

* Within a column, LT values for each exposure time pair with the same letter are not significantly different (*p* > 0.05) according to the lethal dose ratios method [43]. ** All Χ^2^ values fit the model at *p* > 0.05.

**Table 2:** Time causing mortality (MT) 50 and 99 with 95% confidence interval (CI) for *Halyomorpha halys* adults exposed to high temperatures, with probit regression parameters.

Adults’ State	Temperature (°C)	n	MT50 (Minutes) *		95% CI_MT50_ (Minutes)		MT99 (Minutes) *		95% CI_MT99_ (Minutes)		Intercept	SE_Intercept_	Slope	SE_Slope_	Χ^2^ **	df
					Lower	Upper			Lower	Upper						
EXA	47.5	400	8.67	a	7.80	9.54	15.65	b	14.06	18.13	−2.89	0.38	0.33	0.04	33.28	43
ENA	47.5	310	9.69	a	8.53	10.83	17.14	a	15.22	20.39	−3.03	0.48	0.31	0.05	22.14	28
EXA	50.0	400	3.72	a	3.31	4.09	6.10	b	5.50	7.12	−3.64	0.61	0.98	0.15	9.05	34
ENA	50.0	310	4.19	a	3.53	4.90	7.67	a	6.40	11.18	−2.80	0.72	0.67	0.17	2.43	28

* Within a column, MT values for each exposure temperature pair (i.e., 47.5 or 50.0 °C) with the same letter are not significantly different (*p* > 0.05) according to the lethal dose ratios method [43]. ** All Χ^2^ values fit the model at *p* > 0.05.

**Table 3:** Effects of *Halyomorpha halys* weight on tested variables and their interactions. Significant *p*-values are indicated in bold. The × is used to indicate the interaction between sources of variation.

Source of Variation	15 min			30 min			60 min		
	F Value	df	*p*-Value	F Value	df	*p*-Value	F Value	df	*p*-Value
Status (dead or alive)	0.10	1; 210	0.7517	0.38	1; 210	0.5404	0.17	1; 210	0.6850
Temperature (Temp)	3.47	1; 210	0.0639	2.47	1; 210	0.1173	1.20	1; 210	0.2739
Status × Temp	0.50	1; 210	0.4804	0.66	1; 210	0.4173	0.00	1; 210	0.9954
**Sex**	**92.77**	**1; 210**	**<0.0001**	**54.81**	**1; 210**	**<0.0001**	**46.79**	**1; 210**	**<0.0001**
Status × Sex	0.31	1; 210	0.5793	1.47	1; 210	0.2273	0.27	1; 210	0.6033
Sex × Temp	0.46	1; 210	0.4968	0.27	1; 210	0.6066	0.83	1; 210	0.3621
Status × Sex × Temp	0.14	1; 210	0.7090	0.73	1; 210	0.3950	0.18	1; 210	0.6732
**Overwintering state (OS)**	**22.12**	**1; 210**	**<0.0001**	**13.88**	**1; 210**	**0.0003**	**16.35**	**1; 210**	**<0.0001**
Status × OS	0.08	1; 210	0.7759	0.41	1; 210	0.5202	0.30	1; 210	0.5815
OS × Temp	0.11	1; 210	0.7391	0.45	1; 210	0.5037	1.29	1; 210	0.2579
Status × OS × Temp	0.51	1; 210	0.4763	0.21	1; 210	0.6442	1.86	1; 210	0.1744
Sex × OS	0.76	1; 210	0.3844	1.84	1; 210	0.1765	1.37	1; 210	0.2439
Status × Sex × OS	0.07	1; 210	0.7867	0.18	1; 210	0.6746	0.62	1; 210	0.4324
Sex × OS × Temp	0.93	1; 210	0.3366	0.69	1; 210	0.4071	0.24	1; 210	0.6227
Status × Sex × OS × Temp	0.03	1; 210	0.8633	0.27	1; 210	0.6029	0.01	1; 210	0.9118

**Table 4:** Effects of *Halyomorpha halys* nutrient index on tested variables and their interactions. Significant *p*-values are indicated in bold. The × is used to indicate the interaction between sources of variation.

Source of Variation	15 min			30 min			60 min		
	F Value	df	*p*-Value	F Value	df	*p*-Value	F Value	df	*p*-Value
Status (dead or alive)	0.14	1; 210	0.7120	0.00	1; 210	0.9877	0.69	1; 210	0.4066
Temperature (Temp)	1.94	1; 210	0.1652	2.55	1; 210	0.1118	0.98	1; 210	0.3223
Status × Temp	0.16	1; 210	0.6930	0.84	1; 210	0.3595	0.02	1; 210	0.9024
**Sex**	1.34	1; 210	0.2481	2.87	1; 210	0.0915	3.88	1; 210	0.0501
Status × Sex	0.03	1; 210	0.8519	1.65	1; 210	0.1999	1.93	1; 210	0.1657
Sex × Temp	0.94	1; 210	0.3343	1.42	1; 210	0.2353	0.28	1; 210	0.6002
Status × Sex × Temp	0.67	1; 210	0.4144	0.17	1; 210	0.6766	0.13	1; 210	0.7237
**Overwintering state (OS)**	**22.5**	**1; 210**	**<0.0001**	**17.77**	**1; 210**	**<0.0001**	**20.34**	**1; 210**	**<0.0001**
Status × OS	0.02	1; 210	0.8758	0.90	1; 210	0.3431	1.03	1; 210	0.3110
OS × Temp	0.29	1; 210	0.5913	0.81	1; 210	0.3701	0.49	1; 210	0.4832
Status × OS × Temp	0.32	1; 210	0.5732	0.62	1; 210	0.4313	0.19	1; 210	0.6646
Sex × OS	0.38	1; 210	0.5361	2.70	1; 210	0.1016	1.80	1; 210	0.1814
Status × Sex × OS	0.01	1; 210	0.9237	0.42	1; 210	0.5154	0.28	1; 210	0.5991
Sex × OS × Temp	3.88	1; 210	0.0501	1.80	1; 210	0.1817	0.53	1; 210	0.4692
Status × Sex × OS × Temp	0.00	1; 210	0.9530	0.00	1; 210	0.979	0.46	1; 210	0.5000
